# Association of 15-hydroxyprostaglandin dehydrogenate and poor prognosis of obese breast cancer patients

**DOI:** 10.18632/oncotarget.15280

**Published:** 2017-02-11

**Authors:** Ruxing Wu, Tao Liu, Peiwen Yang, Xiyou Liu, Fei Liu, Ya Wang, Huihua Xiong, Shiying Yu, Xiaoyuan Huang, Liang Zhuang

**Affiliations:** ^1^ Department of Obstetrics and Gynecology, Tongji Hospital, Tongji Medical College, Huazhong University of Science and Technology, Wuhan, China; ^2^ Department of Pediatrics, Aflac Cancer Center and Blood Disorders Service, Emory University School of Medicine, Atlanta, GA, USA; ^3^ Cancer Center, Tongji Hospital, Tongji Medical College, Huazhong University of Science and Technology, Wuhan, China

**Keywords:** HPGD, breast cancer, obese, prognosis

## Abstract

In order to explore the new mechanism that obesity worsens the prognosis of breast cancer, we reanalyzed the data about gene expression of normal, overweight, and obese breast cancer patients to explore potential genes and validate its function by clinical and experimental data. The fold change of 15-hydroxyprostaglandin dehydrogenate (HPGD) gene which displayed declining trend with BMI increase was 0.46 in obese versus normal weight patients. HPGD protein was highest expressed in normal weight group and lowest expressed in obese group. The rate of positive lymph nodes was 67% in low expression of HPGD group and 35% in high expression of HPGD group. The recurrence-free survival (RFS) rate and overall survival (OS) rate of 5 years had significant difference between low expression of HPGD group and high expression of HPGD group. Obesity dramatically decreased the RFS rate and OS rate of 5 years. Down regulation of HPGD expression could increase the migration and proliferation ability of breast cancer cell line MCF-7. Taken together, our results indicate that low expression of HPGD may be a reason for poor prognosis of obese breast cancer patients.

## INTRODUCTION

Breast cancer accounts for 23% of all cancer cases and is the common cause of cancer death [1]. Females with body mass index (BMI) increased are found to be more risk of developing breast cancer [2]. An increased recurrence rate and a decreased survival rate in breast cancer is consistently related to obesity [3–5].

The mechanism of how obesity affects the prognosis of breast cancer is complex. It's well known that obese women have hyperinsulinemia or insulin resistance, and whose adipose tissues secrete adiponectin, estrogen, leptin, and incalculable less well-characterized epithelial cell mitogen. These factors may stimulate breast tumor evolution [6, 7]. However, its exact mechanism remains unclear. To further study the correlation between BMI and breast cancer, Creighton et al. have evaluated gene expression of normal, overweight, and obese breast cancer patients and suggested that obesity down-regulated or up-regulated the gene expression patterns of breast cancer patients [8].

To verify which genes are associated with poor prognosis of obese breast cancer patients, we reanalyzed the data provided by Creighton et al. from National Center for Biotechnology Information Search database (NCBI) [8]. The 15-hydroxyprostaglandin dehydrogenate (HPGD) gene that met the criterions was certificated by immunohistochemistry (IHC). Furthermore, we followed up the breast cancer patients who underwent the reasonable treatment to confirm the significance of HPGD. We also explore the correlation between down regulation of HPGD expression and the migration and proliferation ability of breast cancer cell line MCF-7.

## RESULTS

### HPGD gene was found by bioinformatics analysis

**Figure 1:**
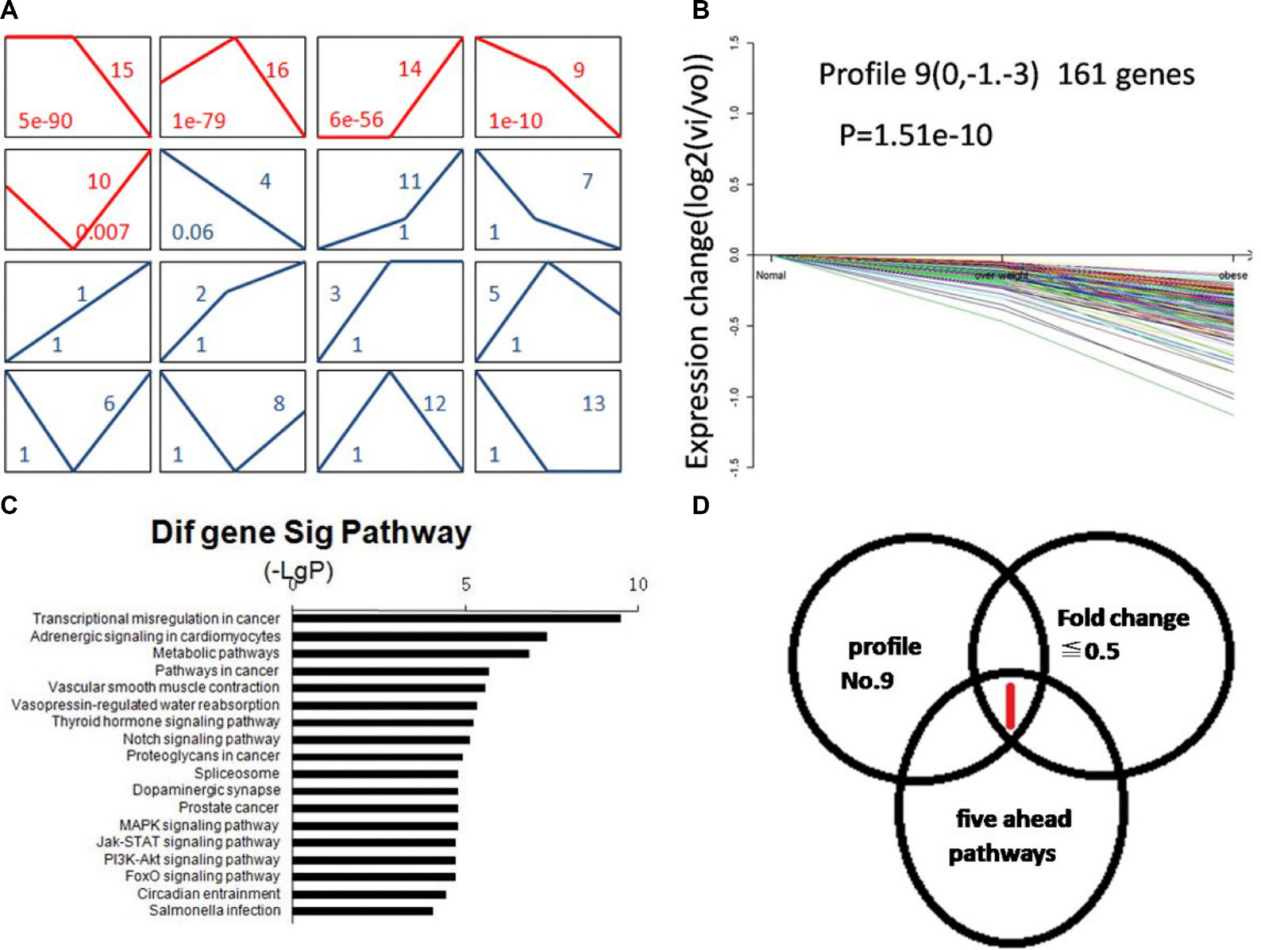
The target gene HPGD was selected by bioinformatics analysis (**A**) The expression patterns of 1,985 DEGs analyzed by 16 model expression profiles. Sixteen expression profiles were defined by cluster analysis to summarize the expression pattern of the1,985 DEGs. Each box represents a model expression profile. The upper number in the profile box is the model profile number and the lower shows the *p-value*. Five boxes of red solid line expression patterns of genes showed significant *p*-values (*p* < 0.05). (**B**) Gene expression of profile No.9. The profile No.9 contained 161 genes and they decreased constantly in expression. The horizontal axis represents BMI and the vertical axis shows gene expression levels after Log normalized transformation. (**C**) Pathway analysis based on down regular DEGs in obesity versus normal weight group. Vertical axis represents pathway category and horizontal axis represents negative logarithm of *p*-values of pathways. (**D)** Screen the target gene from DEGs. It only HPGD gene was considered as candidate gene. The left cycle represents fold change ≤ 0.5 of genes, the right cycle represents profile NO.9 genes and the lower cycle represents the five ahead significant pathways of down regular genes in obesity versus normal weight group genes.

A total of 1,985 differentially expressed genes (DEGs) were identified among the three groups. We further chose16 expression profile patterns to summarize the1,985 DEGs by cluster analysis. Among the 16 patterns, we identified 5 patterns of red solid line that showed significant differences (*p* < 0.05) (Figure 1A). Since profile No. 9 has declining trend pattern from normal weight to overweight to obese group (Figure 1B). We combined the genes with the fold change ≤ 0.5, in profiles No. 9, and in the five ahead significant pathways of down regular genes in obese versus normal weight group to screen the DEGs (Figure 1C, 1D). Thus, only 15-hydroxyprostaglandin dehydrogenate (HPGD) gene, of which the fold change was 0.46, was considered as candidate gene and in the “Transcriptional misregulation in cancer” pathway.

### Expression of HPGD protein decreases in cancer tissues and obese patients by IHC

**Figure 2:**
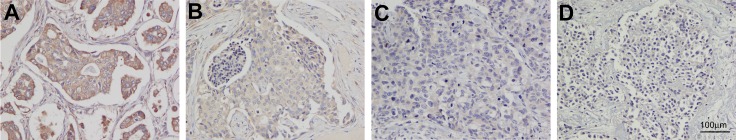
Human primary breast cancer tissues were immunostained with HPGD antibody Immunohistochemical score was based on staining intensity and percentage of HPGD positive cells. Typical images of intensity grades are presented (A = strong positive; B = positive; C = weak; D = negative). Original magnification of (**A**–**D**) is 400×. Scale bar represents 100 microns.

Clinical and pathologic characteristics had no significant difference among normal weight group, overweight group and obese group (Table 1). Figure 2 was the typical stains with stronger positive, positive, weak positive and negative expression of HPGD protein in cancer tissues, respectively. Immunohistochemical scores of cancer tissues in different BMI groups were displayed in Table 2A. The rate of high expression of HPGD protein in breast cancer tissues was 70.2%, 56.2% and 35.5% in normal weight, overweight and obese patients, respectively. The rate of high expression of HPGD protein in adjacent normal tissues was 90.5%, 86.3% and 75.8% in normal weight, overweight and obese patients, respectively.

**Table 1:** Clinical and pathologic characteristics of breast cancer patients receiving follow up and their samples immunostained

Characteristics	Normal Weight(*N* = 84)	Overweight(*N* = 73)	Obesity(*N* = 62)	χ^2^	*p* Value
Age				1.222	0.543
≤ 40	26	17	16		
> 40	58	56	46		
Menopause				0.895	0.639
post-	24	26	20		
pre-	60	47	42		
Tumor Size				0.998	0.607
≤ 2 cm	36	35	31		
> 2 cm	28	38	31		
Lymph Node				3.3	0.192
Yes	36	36	36		
No	48	37	26		
Surgery				3.911	0.141
Modified- radical	29	27	31		
radical	55	46	31		
Histology				0.638	0.727
invasive	73	66	56		
noninvasive	11	7	6		
Grade				7.424	0.115
I	23	18	9		
II	43	34	28		
III	18	21	25		
ER				4.202	0.122
positive	51	38	43		
negative	33	35	19		
PR				1.159	0.56
positive	46	45	33		
negative	38	28	29		
HER-2				2.848	0.241
positive	20	13	8		
negative	64	60	54		
Radiotherapy				1.353	0.508
Yes	37	33	33		
No	47	40	29		
Endocrine Therapy				2.117	0.347
Yes	8	10	11		
No	76	63	51		
Neoadjuvant Chemotherapy				4.493	0.082
Yes	5	9	11		
No	79	64	51		

**Table 2A:** Immunohistochemical scores of cancer tissues in different BMI group

BMI	Low Ex-(*N* = 97)	High Ex- (*N* = 122)
Normal Weight	25	59
Overweight	32	41
Obesity	40	22

**Table 2B:** Difference of immunohistochemistry scores of cancer tissues in each group

group	χ^2^	*p* Value
obesity vs normal weight	19.079	0.000
overweight vs normal weight	4.204	0.04
obesity vs overweight	5.761	0.016

### Expression of HPGD protein decreases in cancer tissues

Statistical difference of the expression of HPGD protein between adjacent normal tissues and cancer tissues was significant in normal weight group (χ^2^ = 10.898, *p* = 0.001), overweight group (χ^2^ = 16.178, *p* < 0.001) and obese group (χ^2^ = 20.422, *p* < 0.001).

### Expression of HPGD protein decreases in obese patients

Statistical difference of the expression of HPGD protein in breast cancer tissues was detected between normal weight group and obese group (χ^2^ = 19.079, *p* < 0.001), between overweight group and obese group (χ^2^ = 5.761, *p* = 0.016), and between normal weight group and overweight group (χ^2^ = 4.204, *p* = 0.04) (Table 2B). Statistical difference of the expression of HPGD protein in adjacent normal tissues between normal weight group and obese group was significant (χ^2^ = 5.784, *p* = 0.016). It showed that the expression of HPGD protein both in cancer tissues and in adjacent normal tissues displayed a gradual decrement trend in normal weight, overweight and obese patients.

### Low expression of HPGD protein, high positive rate of lymph nodes

There were no significant difference in age, whether menopause, tumor size, histology, clinical grade, ER, PR and HER-2 status between high expression of HPGD protein group and low expression of HPGD protein group (Table 3). The rate of positive lymph nodes patients was 67 % and 38% in low expression of HPGD protein group and high expression of HPGD protein group respectively, of which the difference was significant (χ^2^ = 21.819, *p* < 0.001). Our results showed that the rate of positive lymph nodes patients was higher in low expression of HPGD protein group in each BMI group and vice versa (*p* < 0.05) (Table 4).

**Table 3:** Clinical and pathologic characteristics of patients whose cancer tissues low or high express HPGD protein

Characteristics	Low Ex-(*N* = 97)	High Ex-(*N* = 122)	χ^2^	*p* Value
Age			0.328	0.567
≤ 40	28	31		
> 40	69	91		
Menopause			0.342	0.559
post-	29	41		
pre-	68	81		
Tumor Size			0.353	0.553
≤ 2 cm	43	59		
> 2 cm	54	63		
Histology			0.007	0.934
invasive	87	109		
noninvasive	10	13		
Grade			0.403	0.817
I	24	26		
II	46	59		
III	27	37		
ER			1.541	0.214
positive	54	78		
negative	43	44		
PR			3.774	0.052
positive	62	62		
negative	35	60		
HER-2			1.214	0.27
positive	15	26		
negative	82	96		

**Table 4:** Whether stained lymph node of patients whose cancer tissues low or high express HPGD protein

BMI		Yes	No	χ^2^	*p* Value
Normal Weight	Low Ex-	16	9	6.497	0.011
	High Ex-	20	39		
Overweight	Low Ex-	21	11	6.064	0.014
	High Ex-	15	26		
Obesity	Low Ex-	28	12	6.595	0.01
	High Ex-	8	14		
Total	Low Ex-	65	32	21.819	< 0.001
	High Ex-	43	79		

### RFS rate of 5 years and survival rate of 5 years

In the 219 patients, there were 47patients relapsed and 27patients died for breast cancer in 5 years. The overall relapse rate of 5 years was 21.46% and the mortality rate of 5 years was 12.32%. There were 29 relapsed patients and 18 died patients in low expression HPGD protein group, the number was18 and 9 in high expression HPGD protein group. There were 13, 15and 19 relapsed patients and 7, 8 and 12 died patients in normal weight, overweight and obese patients, respectively.

### Lower expression of HPGD protein in cancer tissues, higher relapse rate of 5 years and death rate of 5 years

**Figure 3:**
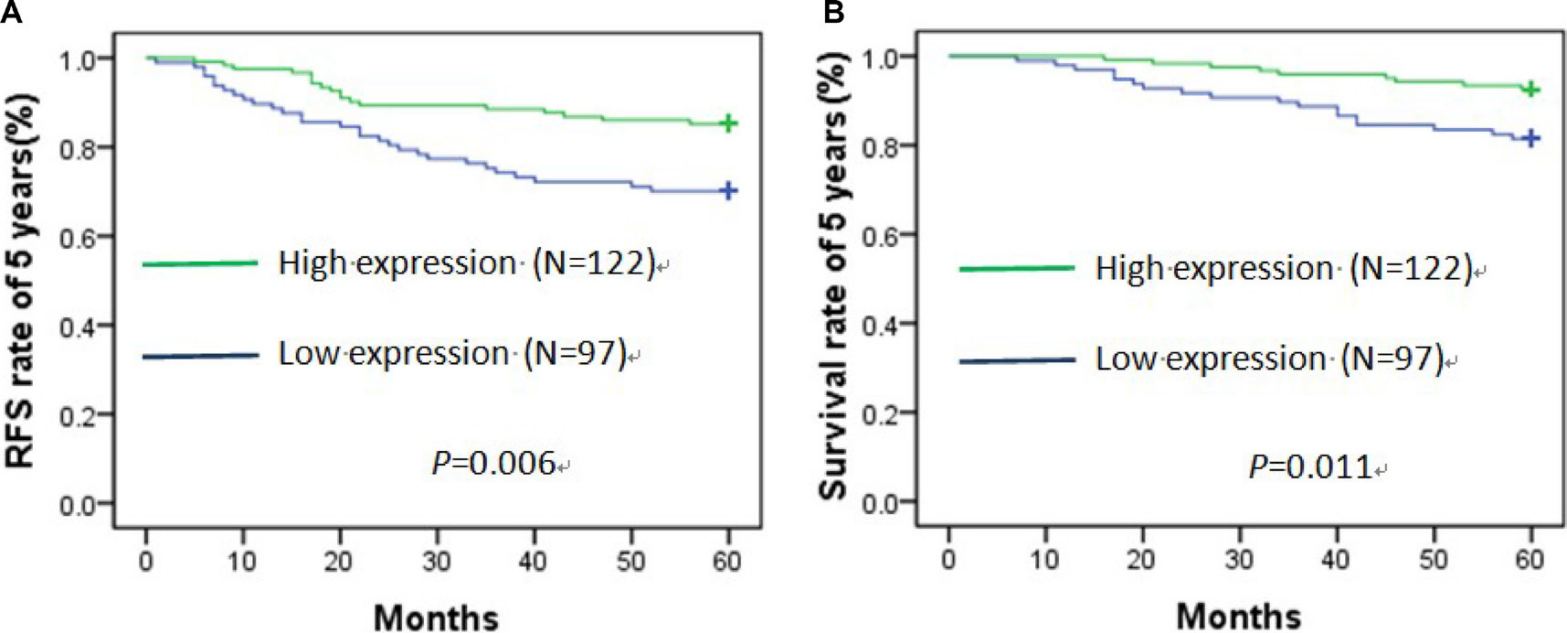
Recurrence-free survival (**A**) and overall survival (**B**) curve of 5 years about low and high expression of HPGD protein in cancer tissues by Kaplan–Meier analysis.

The recurrence-free survival (RFS) rate of 5 years was 85% in high expression of HPGD protein group and 70% in low expression of HPGD protein group, and statistical difference was significant (χ^2^ = 7.641, *p* = 0.006) (Figure 3A). The overall survival (OS) rate of 5 years was 92.6% in high expression of HPGD protein group and 81.4% in low expression of HPGD protein group, and it also had significant difference (χ^2^ = 6.455, *p* = 0.011) (Figure 3B). We further did univariate and multivariate analysis for death and recurrence of 5 years by Cox regression model (Table 5). With unadjusted HR of 2.362 (95%CI, 1.308–4.265), there was a close relationship between HPGD and breast cancer relapse. After adjusting for other positive predictors, HPGD still maintained an independent relapse predictor with an adjusted HR of 2.85 (95% CI, 1.38–4.01). In addition, surgical method and HER-2 status remained significant relapse predictors. Similarly, with an unadjusted HR of 2.236 (95% CI, 1.024–4.884), HPGD had a strong relationship with death. Furthermore, HPGD also had robust predictive ability with an adjusted HR of 2.324 (95% CI, 1.063–5.081) after adjusting with other positive factors. In addition, our data showed HER-2 status was a significant death predictor as well.

**Table 5:** Univariate and multivariate analysis for death and recurrence of 5 years by Cox regression models

Parameter	Univariate Analysis			Multivariate Analysis		
	HR	95% CI	*p*	HR	95% CI	*p*
**Predictor: recurrence of 5 years**						
Age (> 40)	1.11	0.576–2.139	0.754	−		
Menopause (post- vs pre)	1.245	0.687–2.257	0.47	−		
Tumor Size (≤ 2cm)	1.127	0.636–1.996	0.682	−		
Lymph Node (Yes vs No)	1.752	0.973–3.155	0.062	−		
Grade (I vs II)	1.135	0.461–2.793	0.783	−		
Grade (II vs III)	1.81	0.879–3.727	0.107	−		
Surgery (MRM vs BCS)	0.49	0.276–0.871	**0.015**	0.472	0.265–0.839	**0.011**
Histology (invasive vs noninvasive)	1.245	0.447–3.47	0.675	−		
ER (negative vs positive)	1.009	0.563–1.807	0.976	−		
PR (negative vs positive)	0.961	0.539–1.714	0.893	−		
HER-2 (negative vs positive)	0.473	0.253–0.884	**0.019**	0.429	0.229–0.804	**0.008**
Radiotherapy (Yes vs No)	1.418	0.798–2.519	0.234	−		
HPGD (Low ex- vs High ex-)	2.195	1.219–3.953	**0.009**	2.362	1.308–4.265	**0.004**
**Predictor: death of 5 years**						
Age (> 40)	1.314	0.53–3.255	0.556	−		
Menopause (post- vs pre)	1.067	0.479–2.375	0.874	−		
Tumor Size (≤2 cm)	1.742	0.808–3.754	0.156	−		
Lymph Node (Yes vs No)	2.139	0.961–4.761	0.063	−		
Grade (I vs II)	1.798	0.571–5.665	0.316	−		
Grade (II vs III)	1.897	0.689–5.219	0.215	−		
Surgery (MRM vs BCS)	0.587	0.276–1.249	0.167	−		
Histology (invasive vs noninvasive)	1.485	0.352–6.269	0.591	−		
ER (negative vs positive)	0.732	0.329–1.628	0.444	−		
PR (negative vs positive)	0.748	0.342–1.633	0.466	−		
HER-2 (negative vs positive)	0.431	0.194–0.96	**0.039**	0.411	0.184–0.916	**0.03**
Radiotherapy (Yes vs No)	1.432	0.671–3.06	0.353	−		
HPGD (Low ex- vs High ex-)	2.236	1.024–4.884	**0.043**	2.324	1.063–5.081	**0.035**

MRM: modified radical mastectomy, BCS: breast-conserving surgery

### Higher BMI, higher relapse rate and death rate of 5 years

**Figure 4:**
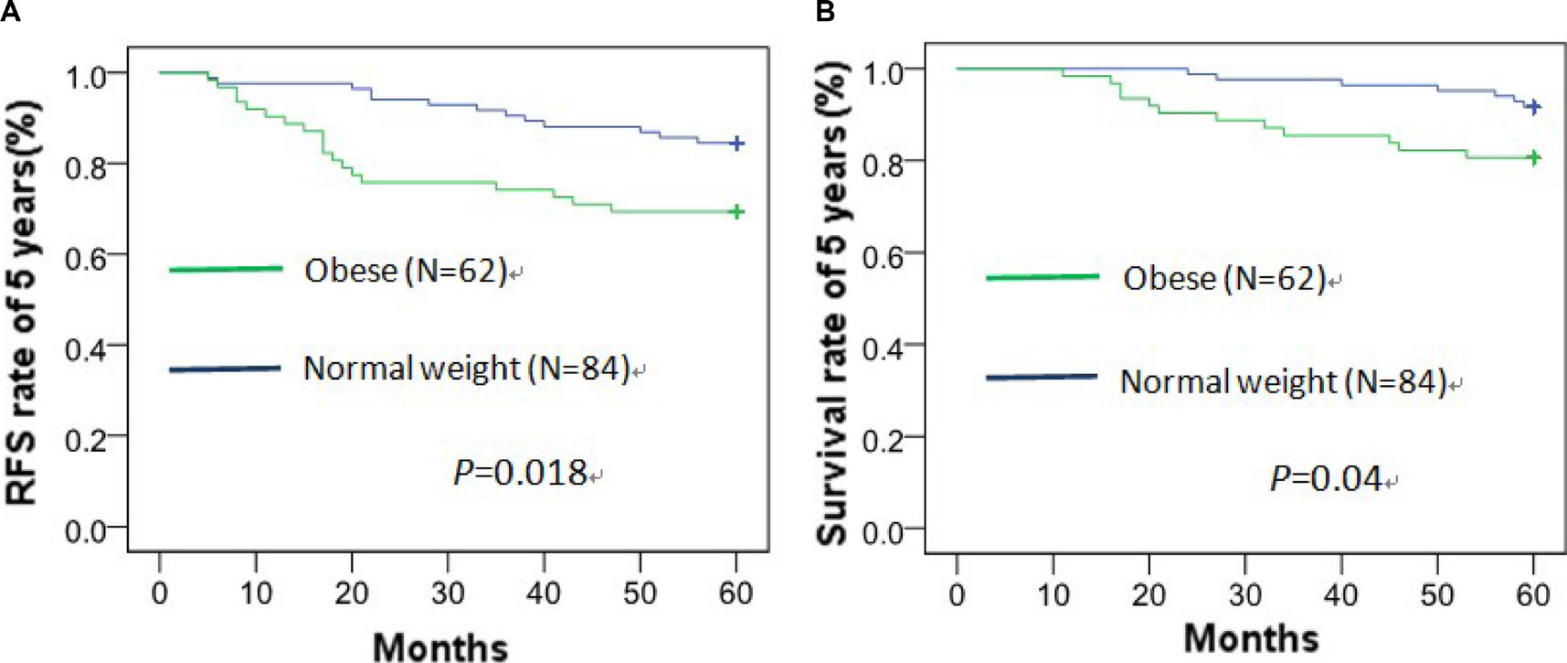
Recurrence-free survival (**A**) and overall survival (**B**) curve of 5 years about obese and normal weight patients by Kaplan–Meier analysis.

The RFS rate of 5 years was 84.5%, 79.5% and 69.4% in normal weight, overweight and obese patients, respectively, and had statistical difference between obese and normal weight patients (χ^2^ = 5.601, *p* = 0.018) (Figure 4A). The OS rate of 5 years was 91.7%, 89.0% and 80.6% in normal weight, overweight and obese patients, respectively, and also had statistical difference between obese and normal weight patients (χ^2^ = 4.226, *p* = 0.04) (Figure 4B).

### Down regulation of HPGD expression could increase the migration and proliferation ability of breast cancer cell line MCF-7

**Figure 5:**
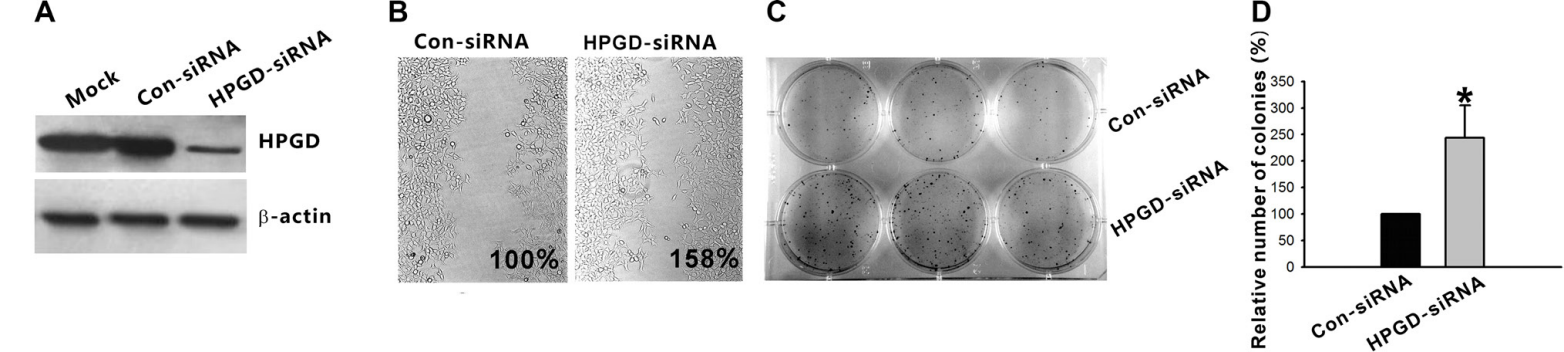
Down regulation of HPGD expression could increase the migration and proliferation ability of breast cancer cell line MCF-7 (**A**) MCF-7 cell lines were transfected with either HPGD-directed siRNA (HPGD-siRNA) or the control siRNA (Con-siRNA). Forty-eight hours after transfection, cells were lysed and immunoblotted with antibody to HPGD, immunoblotting with β-actin antibody was used for loading control. (**B**) Stretch assay showed that HPGD impairment could increase the migration ability of MCF-7 cell lines. Both MCF-7 cell lines with stable silencing of HPGD or stable transfected with Con-siRNA were plated and incubated until confluent. A wound was scratched across each well and wound closure was monitored hourly for 6 h. Wound closure was determined as a percentage of wound confluence compared with the respective negative control (regarded as 100%). (**C**) Two days following transfection with the indicated plasmids (Con-siRNA or HPGD-siRNA), G418 (500 μg/mL) was added to the culture medium, and at day 14, the cells were stained using gentian violet. Untransfected cells were treated similarly, and all died within 2 weeks of culture in the selection medium. Colony formation assays indicated that silencing of HPGD could increase the proliferation ability of MCF-7. (**D**) Quantification was done using AlphaImager 2000. The number of colonies of HPGD-siRNA group compared with the Con-siRNA group (regarded as 100%) was 244%. Columns, mean of three independent experiments; bars, SD; *p < 0.05.

Analyses of 15-PGDH protein expression showed that HPGD-siRNA effectively decreased expression of the gene (Figure 5A). Stretch assay showed that HPGD impairment could increase the migration ability of MCF-7, for which wound confluence of HPGD-siRNA group compared with the Con-siRNA group (regarded as 100%) was 158% (Figure 5B). Colony formation assays indicated that silencing of HPGD could increase the proliferation ability of MCF-7(Figure 5C, 5D).

## DISCUSSION

Our results verified that the expression of HPGD showed a gradual decrement trend in normal weight, overweight and obese breast cancer patients by using bio-information analysis and IHC technology. The expression of HPGD protein decreases in cancer tissues, similarly, Kochel et al. demonstrated that HPGD mRNA expression was decreased in breast cancer compared with healthy breast tissue [9]. Our results also showed that obese patients and low expression of HPGD protein increased the risk of relapse and death after breast cancer, which was consensus with what Kochel et al. reported that high HPGD expression was associated with improved OS and RFS of breast cancer patients [9]. Furthermore, down regulation of HPGD expression stimulated the migration and proliferation ability of breast cancer cell line MCF-7, which was similar to what Wolf et al. had reported [10]. Therefore, these results indicate that low expression of HPGD correlate with the mechanism that obesity worsens the prognosis of breast cancer.

HPGD has been indicated to be a tumor suppressor in lung cancer [11], bladder cancer [12] and colon cancer [13]. The possible mechanisms of low expression of HPGD promoting cancer progression are listed as follows: firstly, HPGD, the prostaglandin E2 (PGE2) metabolizing enzyme, is the key enzyme that regulates prostaglandins (PGs) level by converting them to the corresponding 15-keto derivatives; so it is responsible for the biological inactivation of PGs [14], which are implicated in the initiation and progression of many malignancies [15, 16]; secondly, HPGD has the ability to antagonize the effects of cyclooxygenase-2 (COX-2) and also inhibits angiogenesis *in vivo*.

Interesting, Lehtinen et al. reported that mean expression of HPGD mRNA was higher in normal breast tissues than in breast cancer samples including breast ductal cancer, breast lobular cancer, breast medullary cancer and others, however, that HPGD mRNA was overexpressed in a subset of clinical breast cancers—breast medullary cancer compared to normal breast tissue samples and that high HPGD mRNA expression associated with poor prognosis [17]. Similarly, HPGD expressed differently in breast cancer cell lines [17], for example, Thill et al. suggested that the level of HPGD in a more invasive breast cancer cell line might be even lower [18]. This data suggested a dual role for HPGD in breast cancer. The dual role for HPGD was as well in prostate cancer [17].

Why the expression of HPGD is reduced in obese breast cancer patients? Hong et al. provided epidemiological evidence that chronic inflammation might mediate the association between obesity and breast cancer [19]. Agresti et al. also reported that serum C-reactive protein(CRP), a sensitive marker of systemic inflammation, was higher in overweight or obese breast cancer [20]. Similarly, Wulaningsih et al. suggested that CRP was associated with mortality from breast cancer [21]. A chronic inflammatory related to obesity can cause oxidative damage and inactivate proteins involved in DNA repair or apoptotic control, resulting in cancer cell initiation and growth [22]. These studies prompted that poor prognosis of obese breast cancer patients was partly due to inflammation induced by obesity. PGs were responsible for a wide variety of biological responses and associated with chronic inflammation [23]. Denkert et al. declared that PGs were implicated in the initiation and progression of breast cancer [15], while HPGD was responsible for the biological inactivation of PGs[14]. Based on the above evidence, it is reasonable to infer that there are strong relationship among obesity, inflammation and HPGD.

BMI at diagnosis was a statistically significant prognostic factor in women with early-stage breast cancer [24, 25]. Recently, Karatas et al. declared that obesity was an independent prognostic factor of decreased pathological complete response to neoadjuvant chemotherapy in breast cancer patients [26]. Recent studies have suggested that higher mortality in obese women might because that obese women were more risk to develop high-grade or hormone receptor–negative cancer [27, 28]. Some subjects have stated that treatment factors mediated the relationship between body weight and cancer outcomes since obese patients had often received less aggressive or dose-reduced therapy [29, 30]. Our results came up with a new mechanism why higher mortality in obese breast cancer patients.

It's worth noting: firstly, the classification of normal weight, overweight and obese groups was according to the BMI at the time of diagnosis while Caan et al. declared that weight gain had no disadvantage on mortality for the first 5 years following breast cancer diagnosis [31], and one meta-analysis revealed that weight loss did not have any advantage on the prognosis of breast cancer [32]; secondly, this study was a retrospective study, the number of sample was small and the follow-up time was short to some extent. Thus, it is optimal to design a randomized study detaching pathological type of breast cancer to verify the correlation between HPGD and poor prognosis of obese breast cancer patients.

## MATERIALS AND METHODS

### Bioinformatics analysis

In this study, the original data (GSE24185) was recovered from Gene Expression Omnibus (GEO) data base. Random variance model (RVM) *t-test* was applied to filter the differentially expressed genes (DEGs) for the control and experiment group. The Hierarchical Clustering tab was used to perform hierarchical clustering on our data. Gene ontology (GO) analysis was applied to analyze the main function of the DEGs [33, 34]. Pathway analysis was detected by Kyoto Encyclopedia of Genes and Genomes (KEGG), Biocarta and Reatome [35–37]. The series test of cluster (STC) algorithm of gene expression dynamics was detected by RVM corrective ANOVA.

### Clinical material

Breast cancer samples were obtained from pathology department of TongJi Hospital, TongJi Medical College, HuaZhong University of Science and Technology between January 2008 and December 2010. The scientific use of tissue materials was permitted by the local ethical committee. All the patients provided informed consent for their information to be used for research. The classification of BMI was according to the established Chinese criteria (normal weight:18.5 ≤ BMI < 24; overweight: 24 ≤ BMI < 28; obesity: ≥ 28) [38, 39].

Included criteria:(1) patients were older than eighteen years;(2) patients were suffered from modified radical mastectomy or breast conserving surgery in our hospital; (3) patients completed stand adjuvant chemotherapy or neoadjuvant chemotherapy in our hospital;(4) patients were not relapse or death before adjuvant chemotherapy and (or) radiotherapy; (5) patients with stage IV of breast cancer were excluded. There were 728 patients met the criteria.

We followed the 728 patients by telephone and their outpatient records in November, 2015. The content of the follow-up contained whether relapse or death, location and time of relapse, time of death and cause of death. We obtained 588 patients’ data in the last. The 588 patients had reexamined every 3 months during the first 2 years after chemotherapy, and every 6 months since the 3rd year, and underwent mammography, whole-body ultrasonic detection(ultrasonography of breasts, axillary fossa, cervical parts, abdomen, and pelvis) and X-ray examination for chest annually [40]. Recurrence events were defined as the occurrence of locoregional relapse(including relapse in chest wall, the ipsilateral breast, local skin and operative scar, internal mammary, ipsilateral axillary nodes and supraclavicular) and/or distant metastasis (bones, lung, liver, brain, contralateral breast cancers et al.).

Two clinical end points were set: RFS rate of 5 years and OS rate of 5 years. The RFS time was defined as the interval between primarily surgical treatment and occurrence of the earliest relapse in 5years. The OS time was defined as the interval between primarily surgical treatment and the date of death in 5years.

### Immunohistochemistry

We randomly selected 84, 73 and 62 patients from the followed 588 patients according to BMI levels, respectively. The breast cancer tissues and adjacent normal tissues were stained with HPGD protein polyclonal antibody (1: 200, A6926; ABclonal, USA). The protocol for IHC has been described in detail in previous Publications [41]. HPGD protein immunohistochemical score was independently evaluated in a blinded fashion based on the staining intensity and the percentage of positive cells, resulting in scores of 0–7. Scores of 0–4 were considered as low HPGD protein expression and scores of 5–7 were considered as high HPGD protein expression [17].

### Cells culture

Human breast cancer cell line MCF-7 was obtained from the China Center for Type Culture Collection (CCTCC, Wuhan, China), cells were grown in Dulbecco's modified Eagle's medium (DMEM, Invitrogen, USA) supplemented with 10% fetal bovine serum (Invitrogen, USA), 50 U/mL penicillin, and 50 μg/mL streptomycin. All cells were maintained in a humidified 37°C incubator with 5% CO2, fed every 2~3 days with complete medium (contain 10% FBS). Lipofectamine 2000 (Invitrogen, USA) was used in all transfections. Stable clones were generated by selection in complete culture medium containing 500 μg/mL G418 (Sigma, USA).

### Small interfering RNA

The primers for HPGD-directed small interfering RNAs (siRNA), directed against bases 69 to 97 of the human HPGD, were designed as the reference [10]. The PCR product was inserted into the pCR2.1 vector (Invitrogen, USA). A scrambled siRNA was designed by the same method and used as a control (control siRNA).

### Western blot analysis

Cells were harvested and lysed for total protein extraction in a buffer containing 50 mmol/L Tris-Cl (pH 7.4), 150 mmol/L NaCl, and 2% NP40 together with a protease inhibitors cocktail (Roche Diagnostics, Switzerland). Approximately 50 μg of protein extracts were loaded on 4% to 15% polyacrylamide gels (Bio-Rad, USA), separated electrophoretically, and blotted from the gel onto polyvinylidene difluoride membrane. The membranes were then immunoblotted with anti-HPGD antibody and anti- β-actin antibody (ABclonal, USA).

### Stretch assays

Both MCF-7 cell lines with stable silencing of HPGD or stable transfected with Con-siRNA were plated and incubated until confluent. A wound was scratched across each well (Wound Maker, Essen BioScience, USA) and wound closure was monitored hourly with Incucyte imaging software (Essen BioScience, USA) for 6 h. Wound closure was determined as a percentage of wound confluence compared with the respective negative control (regarded as 100%).

### Colony formation assays

Two days following transfection with the indicated plasmids, G418 (500 μg/mL) was added to the culture medium, and at day 14, the cells were stained using gentian violet. Untransfected cells were treated similarly, and all died within 2 weeks of culture in the selection medium. Quantification of the results was done using AlphaImager 2000 (Alpha Innotech, USA).

### Statistical analysis

Chi-square (χ^2^) test was used to compare differences among categorical variables (performing Fisher exact test when χ^2^ test was unavailable). RFS and OS curves were determined by the Kaplan–Meier methods, meanwhile comparisons between curves were examined by the log-rank test. The relative prognostic importance of HPGD and some clinical variables on recurrence and death were computed by univariate and multivariate analyses using a Cox regression model which was used to obtain crude and multivariate hazard ratios (HR) after adjusted for other significant predictors. All the statistical analyses and curves were done by SPSS software 22.0 (SPSS, Chicago, IL). A *p value* which was less than 0.05(two-sided) was considered to be significant.
